# Hospitalists: the missing link in complex patient care

**DOI:** 10.3325/cmj.2023.64.374

**Published:** 2023-10

**Authors:** Aleksandar Džakula, Karmen Lončarek, Leif Hass, Dorja Vočanec

**Affiliations:** 1 Department of Social Medicine and Organization of Health Care, Andrija Štampar School of Public Health, University of Zagreb School of Medicine, Zagreb, Croatia; 2 Center for Integrated and Palliative Care, University of Rijeka, Faculty of Medicine, Rijeka, Croatia; 3 Department of Hospital Medicine, Alta Bates Summit Medical Center, Oakland, California

## The evolution of hospitals: specialization and fragmentation

Throughout history, the development of medicine and hospitals has been closely intertwined. As new knowledge and innovative treatment approaches emerged, they were promptly integrated and applied within the hospital setting. Hospitals have evolved from being sanctuaries for the frail and infirm into hubs of novel therapeutic techniques. With each advancement, hospitals have embraced increasingly complex knowledge and techniques leading to the need for specialized institutions, equipment, and professionals. This trend pushed medicine toward high specialization, with institutions and professionals honing in on specific health issues and procedures.

Then, about 25 years ago, a new term emerged: hospitalist. Its introduction did not create a buzz, but it has slowly altered the way doctors work in hospitals. In 1996, Wachter and Goldman published an article advocating for the creation of a new role in hospitals, a kind of “generalist” doctor who would oversee a patient's entire care, understand various medical conditions, and coordinate comprehensive treatment (1). Their vision entailed the systematic cultivation of hospital-based generalists who engage with other members of the care team to comprehend diverse medical conditions, address patients' multifaceted needs, and coordinate comprehensive treatment plans.

“*A hospitalist is a physician who cares for inpatients, meaning they only work inside a hospital. These doctors have often completed residency training in general internal medicine, paediatrics, neurology, obstetrics and gynaecology, or other related fields. They may also be board-certified in hospital medicine. Hospitalists provide timely attention to all your needs, including diagnosis, treatment, and coordination of care across the many specialists you might see during your stay.*” (2)

The introduction of the position of hospitalist was a result of the real needs of patients, but also the necessity for hospitals to care for their patients in a better coordinated, less expensive, and more comprehensive way. After several decades of more specialization, which led to more fragmented care, there was no one who was responsible for the whole person. Health care leaders have recognized the need for the horizontal integration of these separated silos of knowledge and skills (3). Patients are not catalogs of individual diseases but living people with their specific and often complex needs, both medical and non-medical, which should be understood and treated individually. Therefore, quality 21st-century care requires recognizing this problem and creating systems to address it.

## Hospital medicine: a new paradigm and roles

The fragmentation of care transformed hospitals into environments where delivering adequate health care to numerous patients with complex, multifaceted conditions became a formidable challenge. Existing specialists, due to their specific areas of expertise or competencies, found it increasingly difficult to meet these demands. Patients began to suffer the consequences of this medical evolution as they gradually disappeared as persons, with health care professionals narrowing their focus solely on diseases. The result was a declining standard of care for individuals with complex needs, coupled with an escalating strain on hospital work and operations. At the same time, many specialists were forced to handle problems outside their expertise, a situation leading to compromised care for even less complex cases.

The introduction of the hospitalist role marked a turning point. Patients finally received comprehensive care, hospitals optimized their care work flows, and other specialists gained the freedom to engage in their areas of expertise. More than 25 years of existence and successful work of the Society for Hospital Medicine confirm that the concept has come to life. *“SHM has approximately 15,000 members, of whom 83 to 85% are physicians, and most of the rest are hospital medicine nurse practitioners and physician assistants, or residents and medical students. Estimates suggest that 50,000 to 60,000 hospitalists currently practice in the United States.”* (4) As one of the founding figures of this society and a pioneer of the hospitalist movement, Winthrop P. Whitcomb noted, *“We saw a need, and we were lucky to be the ones who addressed it. But I think the biggest legacy is for all the patients who benefitted from having a doctor present in the hospital at their time of need to have a skilled clinician who would show up for them in real-time.”* (4)

## Why did hospital medicine and hospitalists emerge solely in North America?

While hospital medicine has thrived for over a quarter-century, its reach beyond North America remains limited, a situation prompting a logical question: why? The challenges associated with narrow specialization and fragmented care are not unique to North America. The needs of patients, particularly of those with multiple comorbidities or requiring coordinated multidisciplinary care, are remarkably consistent worldwide.

The limited global expansion of hospital medicine can be attributed to various factors, as elucidated in a 2018 paper by Flora Kisuule and Eric Howell (5). The foremost among these factors is the strong emphasis on quality and financial outcomes in US hospitals – a driving force that has incentivized the use of hospitalists to reduce patient stays and costs while improving care. In many countries, the emphasis on efficiency and quality remains underdeveloped, a situation that makes it challenging to determine the impact of hospitalists on costs and outcomes. Consequently, cost-reduction strategies often involve the employment of young doctors or part-timers who lack decision-making autonomy or institutional commitment to be effective in the hospitalist role.

The perception of generalists within the medical profession also plays a significant role. In many countries, doctors specializing in general medicine are undervalued and undercompensated. This societal and professional bias diminishes the autonomy and prospects for development of potential hospitalists, and, consequently, their earning potential. To make the work more rewarding, most hospital systems offer the physicians a bargain they readily accept: you can see fewer patients and spend more time on building a mutually satisfying relationship in exchange for a higher-quality and shorter hospital stay.

In some regions where hospitalist programs have been implemented, inadequate education has been an issue. Conventional medical education does not prepare physicians to be part of care teams and for leadership in cost containment, quality, and safety efforts. Successful hospitalist teams need leaders with dedicated time to support these efforts.

## Hospitalists and complex patients

A particular challenge arises from congestion within health care systems. The high cost of care in hospitals in the US is a potent motivator to develop alternative settings for complex but medically stable patients. In countries lacking well-defined long-term care models, patients often linger in acute or other hospitals for extended periods waiting for appropriate placement after their acute medical issues are addressed. This situation undermines the significance of hospitalists’ role in expediting hospital processes, lowering costs, and improving care quality.

Returning to the title's question – what do hospitalists mean for complex patients? – we find ourselves in a paradox. High-quality care for complex patients requires well-educated, competent hospitalists as part of a team who understand their patients’ medical and social needs. In the absence of such professionals, the needs of complex patients go unrecognized within society and the health care system, posing significant challenges during admission, hospitalization, and discharge. Hospitalization without addressing these needs will likely end with frequent readmissions for the same problem. Therefore, the lack of comprehensive patient care within society and health care perpetuates a climate in which there appears to be no place for hospitalists. On the other hand, the absence of hospitalists further complicates and worsens the condition of complex patients. Clearly, a holistic approach combining hospitalists, organized care for complex patients, and long-term care is needed, provided they function as an integrated, cohesive system, breaking free from the vicious circle of fragmented care.
